# A novel approach to mapping load transfer from the plantar surface of the foot to the walls of the total contact cast: a proof of concept study

**DOI:** 10.1186/1757-1146-5-32

**Published:** 2012-12-13

**Authors:** Lindy Begg, Patrick McLaughlin, Leon Manning, Mauro Vicaretti, John Fletcher, Joshua Burns

**Affiliations:** 1 Foot Wound Clinic, Department of Surgery, Westmead Hospital, PO Box 533, Wentworthville, NSW, 2145, Australia; 2 Univeristy of Sydney, Department of Surgery, Westmead Hospital, PO Box 533, Wentworthville, NSW, 2145, Australia; 3 School of Biomedical and Health Sciences, Faculty of Health, Engineering and Science, Victoria University, Melbourne, 8001, Australia; 4 Institute of Sport, Exercise and Active Living, Victoria University, Melbourne, VIC, 8001, Australia; 5 Institute for Neuroscience and Muscle Research, The Children’s Hospital at Westmead/Paediatric Gait Analysis Service of New South Wales/Faculty of Health Sciences, The University of Sydney, Sydney, NSW, 2145, Australia

**Keywords:** Total contact cast, Offloading, Plantar pressure, Diabetes

## Abstract

**Background:**

Total contact casting is regarded as the gold standard treatment for plantar foot ulcers. Load transfer from the plantar surface of the foot to the walls of the total contact cast has previously been assessed indirectly. The aim of this proof of concept study was to determine the feasibility of a new method to directly measure the load between the cast wall and the lower leg interface using capacitance sensors.

**Methods:**

Plantar load was measured with pedar® sensor insoles and cast wall load with pliance® sensor strips as participants (n=2) walked along a 9 m walkway at 0.4±0.04 m/sec. The relative force (%) on the cast wall was calculated by dividing the mean cast wall force (N) per step by the mean plantar force (N) per step in the shoe-cast condition.

**Results:**

The combined average measured load per step upon the walls of the TCC equated to 23-34% of the average plantar load on the opposite foot. The highest areas of load on the lower leg were located at the posterior margin of the lateral malleolus and at the anterior ankle/extensor retinaculum.

**Conclusions:**

These direct measurements of cast wall load are similar to previous indirect assessment of load transfer (30-36%) to the cast walls. This new methodology may provide a more comprehensive understanding of the mechanism of load transfer from the plantar surface of the foot to the cast walls of the total contact cast.

## Background

A total contact cast (TCC) is a well-moulded, minimally padded cast that maintains contact with the entire plantar aspect of the foot and lower leg [1]. TCC is regarded as the ‘gold standard’ treatment for plantar foot ulcers in people with diabetes [1-3] yet application of the technique varies considerably [4]. The TCC immobilises the limb, reduces oedema and ensures ‘forced compliance’ [1]. Protection from trauma, along with a shorter stride length and velocity during gait are also important attributes of the TCC [5].

In our tertiary hospital foot wound clinic, the TCC technique has evolved commensurate with the development of new materials; from plaster with a plywood base and a walking heel, to fibreglass with cast under-wrap, to a combination of semi-rigid and rigid materials with no under-wrap and using a cast shoe. More recently, this technique was optimised by incorporating an inlay of 6 mm slow-rebound cellular urethane and 6 mm soft cellular urethane [6]. The addition of these cushioning materials significantly reduced peak pressure at the ulcer site by 70%, mean pressure by 60% and pressure–time integral by 69% [6].

The offloading mechanism of a TCC is attributed to the redistribution of weight-bearing pressure across the entire plantar surface of the foot and by increasing the plantar surface contact area [5,7]. However, recent contact area data and regional pressure patterns between conditions [6] suggests this is not entirely the case. Rather, the addition of 6 mm slow-rebound cellular urethane and 6 mm soft cellular urethane inlay seems to act by redistributing pressure *without* increasing the plantar surface contact area [6]. Instead, pressure shifts from high zones to low zones without creating high pressures elsewhere, thereby reducing plantar pressure at the site of ulceration [6].

The other proposed offloading mechanism is by transfer of load to the cast walls of the TCC [8-10]. Various studies have assessed this mechanism by *indirect* methods. Shaw and collaeagues placed a capacitance sensor plantar insole (pedar®, novel gmbh, Germany) in a TCC and asked participants to walk across a force platform [8]. Data were collected simultaneously from both the force platform and the inside of the TCC. The authors reported that the difference in impulse, with the plantar insole reporting smaller values than the force platform, was indicative of the load transferred to the cast wall. This difference was calculated as 31% of the impulse measured by the force plate. Leibner and co-workers [9] asked participants to wear a TCC, followed by a cut-down version of the same TCC referred to as a shoe-cast, during walking trials, and measured plantar load using a capacitance sensor plantar insole (pedar®, novel gmbh, Germany). The smaller values for average force per step in the TCC condition were attributed to a transfer of load to the cast walls compared to the shoe-cast condition. This transfer of load was calculated at 36% of the average force per step measured in the shoe-cast condition. Finally, Tanaka and colleaugues (2000) compared the output from plantar insoles (F-SCAN, Tekscan Inc, South Boston, Massachusetts) in a patella tendon cast (used for the treatment of below knee fractures) and the contralateral side (extension shoe). These authors reported that the difference in plantar load due to the cast walls was 30% [10]. Whilst these authors used similar indirect methods to estimate cast wall load, it is also clear that each author reported different units of measurement making it difficult to assess the level of agreement. However, the reduction of plantar load via transfer to cast walls was in the vicinity of 30%.

No previous research has directly measured the load on the cast walls. Therefore, the aim of this pilot study was to determine the feasibility of a new method to directly measure the load between the cast wall and the lower leg interface using capacitance sensors. Further aims were to determine areas of maximal load between the cast wall and the lower leg and to directly determine load transfer from the plantar surface of the foot to the cast walls.

## Methods

A 20 year old healthy female and a 32 year old female with a 17 year history of Diabetes Mellitus with no history of diabetic foot complications were recruited from a private Podiatry Clinic. The two participants were physically matched, had the same shoe size and indicated a willingness to participate in the study. The participants were provided with informed written consent in accordance with the Human Research Ethics Committee (HREC 2009/12/5.12 (3093).

The TCC method applied to both participants involved a combination of rigid and semi-rigid cast materials, with the addition of 6 mm slow-rebound cellular urethane and 6 mm soft cellular urethane inlay as described previously [6]. After 20 to allow for drying time, as recommended by the manufacturer, each participant walked along a 9m walkway to familiarise themselves with walking while wearing a TCC. The TCC was bi-valved and a capacitance sensor insole with a resolution of 1.2 sensors per cm^2^ (pedar®, novel Gmbh, Germany) was placed on to the plantar area of the TCC and another into the participant’s shoe (Adidas Adistar Ride, Adidas International, Herzogenaurch, Germany) on the non-casted contralateral limb.

Pliance® sensors were *also* placed along the lower leg in order to measure load between the walls of the cast and the lower leg (pliance®, novel Gmbh, Germany). The pliance® sensor consisted of two sets of 3 × 15 sensors, with each capacitance sensor having an area of 1 cm^2^ producing a combined capacitance sensor area of 90 cm^2^ when both sensors were placed on the lower leg. The pliance® sensor is less than 1 mm thick and calibrated to a pressure range of 4-60kPa.

**Figure 1:**
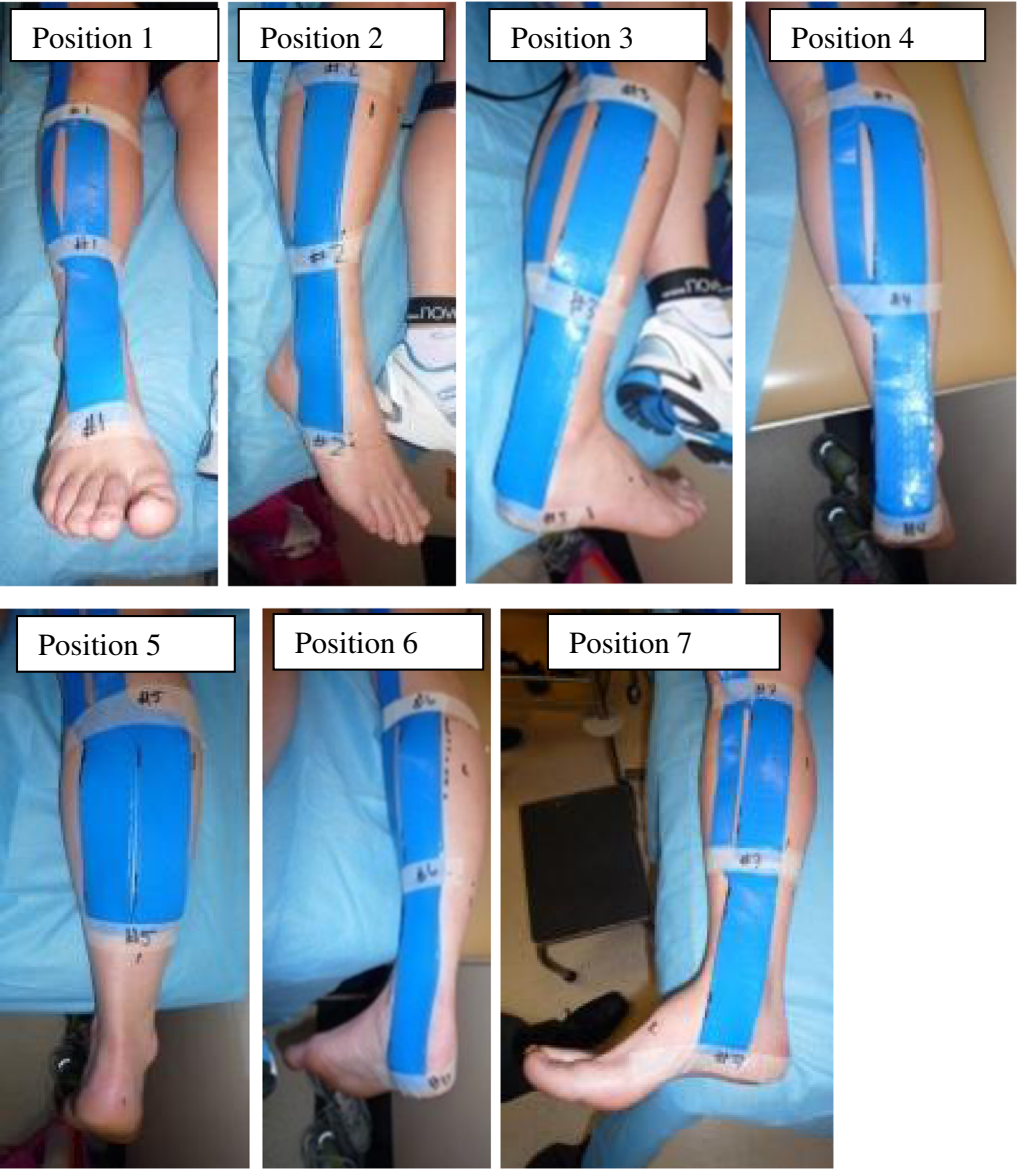
Orientation of sensors used to measure cast wall pressures at the seven different locations.

To assess the feasibility of assessing load over the entire lower leg, it was necessary to open the TCC, relocate the sensors and repeat the measurements. An ink marker was used upon the participant’s skin to outline the sensors to ensure accuracy of relocation and to reduce the risk of overlapping. It was necessary to repeat this process seven times to ensure the systematic coverage of the entire surface area of the lower leg (Figure 1 Position 1-7). Non-stretch strapping tape was used to reaffix the bivalve edges and to return the bi-valved cast to a TCC for data collection.

The participants walked at a comfortable walking speed of approximately 0.4 ± 0.04 m/sec over a 9 m walkway. Trials whereby the participants walked at a velocity outside a 10% tolerance were excluded from the study. Both pedar® and pliance® systems (novel Gmbh, Germany) collected data simultaneously. Sample rate for both systems was 50Hz. In order to illustrate the overall picture of cast wall load, the seven TCC trials were synchronised and combined based on the temporal event of heel strike. Each trial started with the participant standing with two feet together. Each participant was instructed to commence walking after the various data collection systems had commenced recording. In this way, the heel strike of the first step of each trial could be used for synchronisation purposes. This synchronisation was completed manually by combining video, plantar and cast wall data. The data from seven locations of the cast wall were synchronised for comparison at the same temporal events.

**Figure 2:**
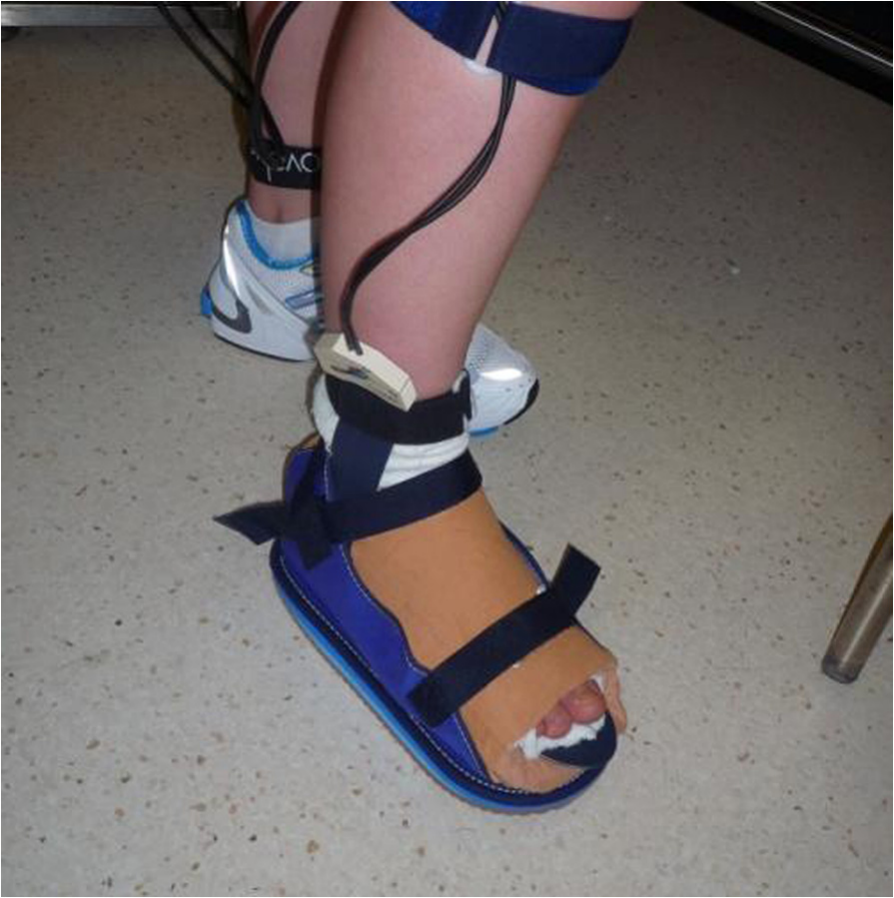
**The shoe-cast condition. ** Cast walls were removed from the TCC leaving a well moulded shoe-cast condition.

After completion of all seven trials measuring each location of the cast wall, the cast wall of the TCC was cut down to create a shoe-cast, as previously described [9] (Figure 2). A canvas cast boot with a slight rocker-sole was added to both the TCC and the shoe-cast, to reduce the likelihood of slipping and tripping.

In summary, for each trial of the TCC; cast wall and plantar load data were collected simultaneously. Plantar force data were collected in the TCC, shoe-cast and the non-casted contralateral limb *i.e.* sport shoe. For each participant, the relative force (%) on the cast wall was calculated by dividing the mean cast wall force (N) per step by the mean plantar force (N) per step in the shoe-cast condition.

## Results

**Figure 3:**
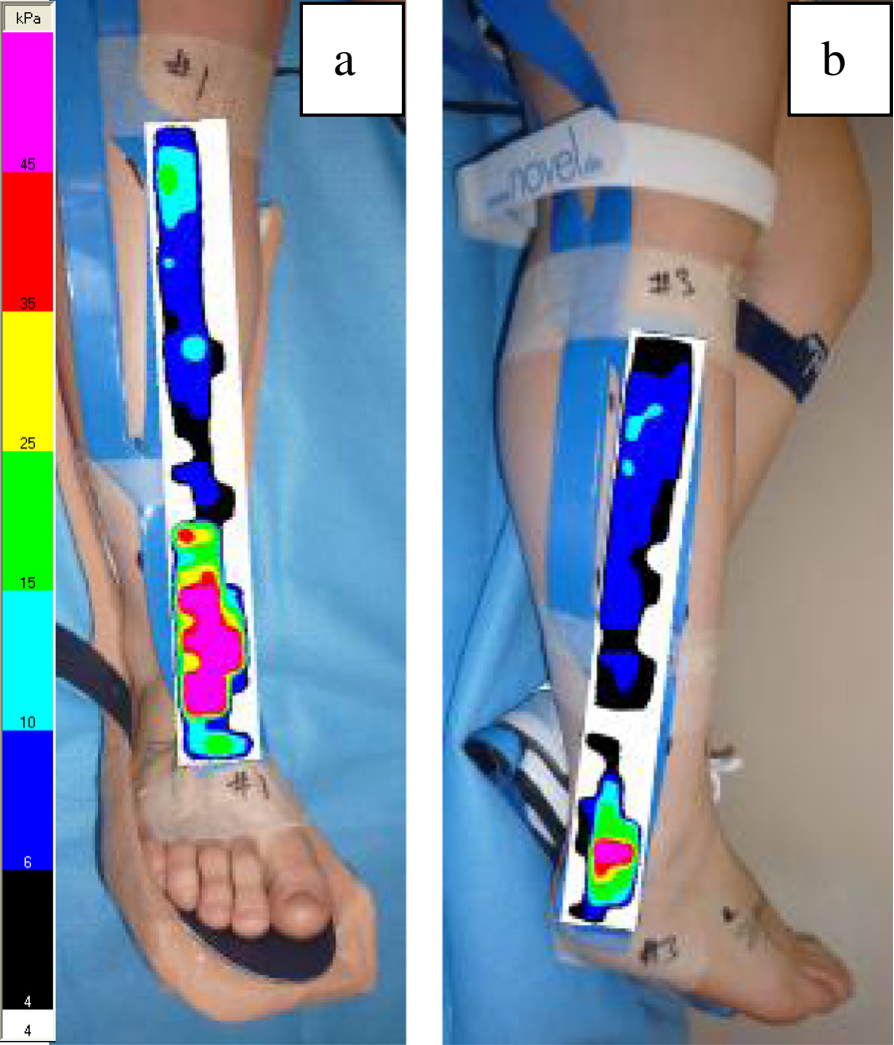
**(a-b) The positions of the cast wall sensors that recorded maximum pressures as indicated by the pink areas. ** Images represent the maximum pressure picture (MPP).

The combined average measured load per step upon the walls of the TCC equated to 23-34% of the average plantar load on the opposite foot (Table 1). Each pliance® sensor indicated that there was load evident between the cast wall and the leg at all locations at different points in the gait cycle. The two highest force locations from the cast-wall pliance® sensors were:

i. Along the tibia, running distally, specifically across the top of the ankle mortise over connective tissue structures (extensor retinaculum), (Figure 3a).

ii. On the postero-lateral part of the lower leg, running distally from a line slightly posterior to the fibula head and passing posterior to the lateral malleolus, specifically at the area of the lateral malleolus (Figure 3b).

**Table 1:** Comparison of plantar and cast wall step force data for each participant in the TCC and shoe-cast conditions

	**Average plantar force/ step (N)**	**Average cast wall force/step (N)**	**Relative cast wall load/step (%)**
**Participant with Diabetes (steps)**	Total foot		Direct method
**Shoe-cast (n=9)**	470±14		34%
**TCC (n=63)**	446±72	159±13	
**Healthy Participant (steps)**			
**Shoe-cast (n=10)**	460±27		23%
**TCC (n=73)**	419±38	104±16	

## Discussion

In this study we were able to directly measure cast wall load using capacitance sensors. The TCC wall received 23-34% of the lower limb load based upon these measurements. These directly measured results appear to confirm previous indirectly calculated cast wall loads of 30-36% [8-10]. This is a finding of interest given the different cast materials utilised in each study. However in each study, casts were similarly applied with the ankle position at 90 degrees in the sagittal plane and that the casts extended proximally to the ankle joint.

Further, the highest areas of load on the lower leg were located at the posterior margin of the lateral malleolus and at the anterior ankle/extensor retinaculum. These two sites are routinely padded to protect bony prominences during casting to minimise the risk of iatrogenic tissue damage. The clinical implications of this study support the concept of offloading modalities that extend proximal to the ankle due to the potential of load sharing from the plantar surface to the walls.

We were required to remove and re-orient the cast wall sensors and manually synchronise data from the different trials. This was a time intensive task that is not feasible in day to day clinical practice. To overcome this, we identified two distinct areas of maximum load between the cast wall and the lower leg and suggest that these areas should be the focus of future measurement studies. Further, it should be noted that this study measured normal, or perpendicular, load and did not measure shear. At the present time, it is not possible to clinically measure shear, therefore clinicians rely upon feedback from patients, and assessment of skin integrity, as indicators of rubbing or friction within the cast. Whilst this is not ideal from a measurement perspective, and is a limitation of this study, it does reflect current clinical practice.

Our new direct measurement approach demonstrated that it is possible to measure the load between the cast wall and the lower leg. However, since this paper has reported the outcome from a proof of concept study; the methodology requires repeating in a larger sample of participants with plantar foot ulceration. Research of this type is necessary to develop a more comprehensive understanding of the TCC offloading mechanism in terms of pressure offloading and the healing of plantar foot ulceration.

## Competing interests

JB receives funds from the NHMRC (National Health and Medical Research Council of Australia, Fellowship #1007569 and Centre of Research Excellence #1031893), NIH (National Institutes of Neurological Disorders and Stroke and Office of Rare Diseases, #U54NS065712), Australian Podiatry Education and Research Foundation, Podiatry Council of New South Wales, Charcot Marie Tooth Association, Muscular Dystrophy Association, CMT Association of Australia. PM is the Australian and New Zealand agent for novel gmbh.

## Authors’ contributions

LB, PM, JB participated in the design of the study and secured funding, LB, PM, LM carried out data collection, PM, JB contributed to statistical analysis. All authors reviewed and approved the final manuscript.
